# Evolution of Nucleotide Punctuation Marks: From Structural to Linear Signals

**DOI:** 10.3389/fgene.2017.00036

**Published:** 2017-03-27

**Authors:** Nawal El Houmami, Hervé Seligmann

**Affiliations:** URMITE, Aix Marseille Université UM63, CNRS 7278, IRD 198, INSERM 1095, IHU – Méditerranée InfectionMarseille, France

**Keywords:** stem-loop hairpin, secondary structure, nucleotide motif, transcription signals, codon-amino acid assignment

## Abstract

We present an evolutionary hypothesis assuming that signals marking nucleotide synthesis (DNA replication and RNA transcription) evolved from multi- to unidimensional structures, and were carried over from transcription to translation. This evolutionary scenario presumes that signals combining secondary and primary nucleotide structures are evolutionary transitions. Mitochondrial replication initiation fits this scenario. Some observations reported in the literature corroborate that several signals for nucleotide synthesis function in translation, and vice versa. (a) Polymerase-induced frameshift mutations occur preferentially at translational termination signals (nucleotide deletion is interpreted as termination of nucleotide polymerization, paralleling the role of stop codons in translation). (b) Stem-loop hairpin presence/absence modulates codon-amino acid assignments, showing that translational signals sometimes combine primary and secondary nucleotide structures (here codon and stem-loop). (c) Homopolymer nucleotide triplets (AAA, CCC, GGG, TTT) cause transcriptional and ribosomal frameshifts. Here we find in recently described human mitochondrial RNAs that systematically lack mono-, dinucleotides after each trinucleotide (delRNAs) that delRNA triplets include 2x more homopolymers than mitogenome regions not covered by delRNA. Further analyses of delRNAs show that the natural circular code X (a little-known group of 20 translational signals enabling ribosomal frame retrieval consisting of 20 codons {AAC, AAT, ACC, ATC, ATT, CAG, CTC, CTG, GAA, GAC, GAG, GAT, GCC, GGC, GGT, GTA, GTC, GTT, TAC, TTC} universally overrepresented in coding versus other frames of gene sequences), regulates frameshift in transcription and translation. This dual transcription and translation role confirms for X the hypothesis that translational signals were carried over from transcriptional signals.

## Introduction

Punctuation marks are inherent to written systems by providing a critical framework for specifying information. Spread along nucleotide sequences, the study of punctuation signals is relatively neglected in genetics and deserves interdisciplinary attention combining molecular biology, linguistics, and coding theory. Involving 64 nucleotide triplets called codons (Elzanowski and Ostell, 2013), the genetic code is a system coding the set of rules by which information is translated from RNA into proteins by living cells and viruses, by specifying which amino acid will be added during protein synthesis. Information encoded within genetic material also possesses superimposed cryptic messages (Popov et al., 1996), revealing highly complex semiotics (e.g., in circular virusoid RNAs, AbouHaidar et al., 2014). The rules of DNA punctuation vary among 25 recognized genetic codes, suggesting these constantly evolve. Codon–amino acid assignment evolved mainly by changes in punctuation codons, namely initiation (start) and termination (stop) codons (Seligmann, 2015b), impacting length and structures of coding and non-coding DNA sequences.

Here analyses focus on the evolution of punctuation signals, assuming two core principles. First, primitive punctuation of nucleotide sequences consists of multidimensional structures (such as stem-loop hairpins) allowing form recognition by DNA and RNA polymerases. This is illustrated by hairpins that are signals formed by self-hybridization of nucleotides indicating where DNA and RNA polymerizations initiate (e.g., in vertebrate mitochondria, Clayton, 1992). Second, we presume that multi- and unidimensional punctuation marks used for nucleotide synthesis were secondarily hijacked for translation. Thus, we propose a multistep model where multidimensional structures later evolved into linear signals, in parallel to book page earmarking using structural recognition, versus memorizing page numbers requiring a consensual code. In this model, we assume that protein synthesis emerged after that of nucleotides.

### From Multidimensional to Unidimensional Punctuation Signals

Hairpins are structural signals spread within genomes of all organisms. In human mitochondria, stem-loop DNA structures define replication origins (Hixson et al., 1986; Clayton, 1992, 2000; Seligmann and Krishnan, 2006; Seligmann et al., 2006a,b; Seligmann, 2008, 2010a, 2011; Seligmann and Labra, 2014). They guide RNA processing in mitochondria (Ojala et al., 1981), and in giant viruses and their virophages (Byrne et al., 2009; Claverie and Abergel, 2009). This ubiquitous structural signaling also applies to RNA:DNA hybrids, which play a role in the origin-independent replication priming in eukaryotic cells (Stuckey et al., 2015) and in transcription termination in human mitochondria (Zheng et al., 2014).

However, different nucleotide sequences can form similar secondary structures. This generates structural ambiguity responsible for versatile and non-specific signals. For example, in prokaryote RNA-based defense systems against genome invasion by parasites (viruses and plasmids), structural ambiguity of dual-RNAs that guide nucleases to degrade invading DNA is used by bacteria possessing orthologous type II CRISPR-Cas defense systems, for which functional exchangeability was recently demonstrated (Fonfara et al., 2014). The dual RNA duplexes consist of hybridization between tracrRNA and crRNA. The tracrRNAs are trans-encoded RNAs that complement crRNAs, short palindromic repeats. Also, analysis of structural RNA similarities between rRNA and RNA viruses unraveled an ancient transition from cellular organisms possessing ribosomes to viruses (Seligmann and Raoult, 2016), which is undetectable when considering only unidimensional (linear) sequence information.

Synthesis of short consensus signals, such as Shine–Dalgarno sequences, is more cost-effective than that of nucleotide sequences forming secondary structures. Hence avoidance of metabolic costs (Akashi and Gojobori, 2002; Seligmann, 2003, 2012b; Brocchieri and Karlin, 2005; Warringer and Blomberg, 2006; Heizer et al., 2011; Chen and Bundschuh, 2012; Raiford et al., 2012; Krick et al., 2014; Chen W.-H. et al., 2016) should favor evolution of linear consensus signals. Consequently, linear signals presumably evolved more recently to become punctuation marks with higher accuracy, specialization, and metabolic efficiency than structural signals.

In some cases, enzymatic recognition requires both primary and secondary structures. In mitochondria, initiation of DNA polymerization requires a short specific sequence close to the 3′ extremity of the light strand replication origin hairpin (Hixson et al., 1986; Clayton, 2003; Wanrooij et al., 2012). We herein propose a three-step model where multidimensional structures later evolved into linear signals.

### Evolution of Origin of Replication: From Structural to Linear Signals

**FIGURE 1:**
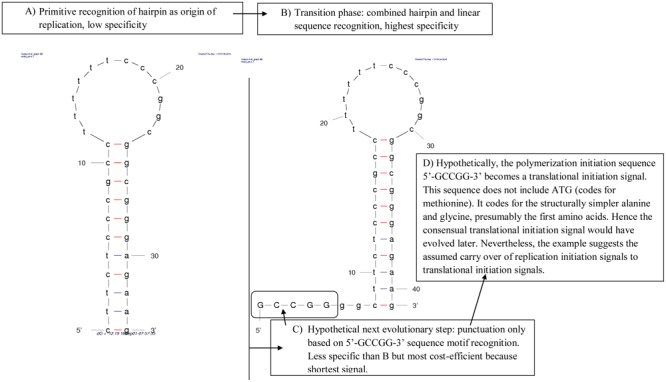
**Hypothetical evolution of the vertebrate mitochondrial light strand replication origin, OL. (A)** The presumed primitive step is polymerization initiated at stem-loop hairpins, such as those formed by DNA templating for tRNAs, which form more OL-like secondary structures in species whose mitogenome lacks a recognized OL. **(B)** DNA replication initiation gains specificity by adding a sequence motif near the hairpin, necessary for initiating polymerization, encountered in most vertebrates. **(C)** The next evolutionary state could be total loss of the stem-loop hairpin, where replication initiation depends solely on a linear sequence motif, potentially losing specificity, but clearly decreasing the length of the sequence required for replication initiation. Observations on replication in taxa lacking OL, such as birds, are compatible with this mechanism. **(D)** The linear motif punctuating polymerization initiation presumably becomes a translational signal. Indeed, 5′-GCCGG-3′ would code for alanine and glycine, the first amino acids integrated in the genetic code according to the consensual order of appearance of organic amino acids.

We illustrate this evolutionary scenario by applying it to the light strand origin of replication (OL) of vertebrate mitochondria (**Figure 1**). In phase A, polymerases only recognize stem-loop hairpins. Indeed, in mitochondria, heavy strand DNA templating for tRNAs form OL-like structures that occasionally function as light strand replication origin (Seligmann et al., 2006b). The polyT sequence in the modern OL loop marks mitochondrial RNA polymerase binding of the OL (Fusté et al., 2010).

Several functional homologies between tRNAs and OL corroborate the hypothesis that tDNA functions as OL. This is in line with functional homologies suggesting that the translation apparatus evolved from DNA/RNA replication (Maizels and Weiner, 1994). First, the OL might function as tRNA because aminoacylated RNA corresponding to the OL was observed (Yu et al., 2008). Second, some tRNAs resemble the OL structure: short armless OL-like tRNAs occur in nematode mitochondria (He et al., 2005; Jühling et al., 2012; Wende et al., 2014). Third, modern tRNAs apparently result from fusion of OL-like tRNA halves (Di Giulio, 2009, 2012a,b, 2013; Branciamore and Di Giulio, 2011). Furthermore, OL-like isolated tRNA arms (Hirose et al., 2015) could have tRNA functions (Seligmann, 2013, 2014): they resemble the OL-like armless nematode tRNAs and their anticodons match genomic codon abundances.

Assuming that **Figure 1A** presents the primitive system, this system acquired higher specificity when combined with recognition of a primary sequence motif, 5′-GCCGG-3′ (**Figure 1B**), which is the typical situation in modern genomes.

In phase B, replication initiation requires both stem-loop hairpin and consensus signal as observed in most vertebrate mitochondria. In phase C, we hypothesize that the structural component of the signal was replaced by a cost-effective consensus linear sequence signal. Hence the signal became less specific by losing the stem-loop hairpin, but less costly in terms of numbers of required constitutive nucleotides, consisting of a shorter linear sequence signal. This evolutionary step could correspond to our incomplete understanding of mitochondrial replication organisms that lack a recognized OL, most birds (Desjardins and Morais, 1991), and some lepidosaurians (Macey et al., 1997; Seligmann and Labra, 2014). Indeed, in bird mitochondria, light-strand replication initiates synchronously at several locations lacking secondary structures (Reyes et al., 2005). Finally, we suggest a hypothetical functional shift from initiation of DNA replication to protein translation (phase D).

### Hijacking of Punctuation Marks from DNA to Protein Synthesis

The principle of evolution from secondary to primary sequence structure punctuation could also be seen at the level of the translational apparatus. Translation involves tRNAs and rRNAs, molecules whose function is inherently based on their multidimensional structure. tRNAs can be interpreted as a combined structural and linear signal, where the anticodon is the linear signal. tmRNAs (transfer-messenger RNA, Janssen and Hayes, 2012; Himeno et al., 2014), which rescue stalled ribosomes on mRNAs lacking proper translational stop signal also combine structural and linear signals, with extremities forming tRNA-like structures, and the rest of the sequence reminding mRNAs (Di Giulio, 2015; Macé and Gillet, 2016). This suggests that tmRNAs are remnants of ancient termination signals. It seems that translation of linear sequence signals (codons) is based on their interplay with multidimensional RNA structures (Brown et al., 2015). This principle applies to dual functions of codons: presence of a stem-loop hairpin on the mRNA determines which amino acid is inserted at the codon, changing the translational signal of the codon (Lobanov et al., 2010).

### From Non-ribosomal Peptide Synthesis to mRNA Translation

Translation by ribosomes is complex and was probably absent at molecular evolution’s first steps (Baranov et al., 2009; Root-Bernstein and Root-Bernstein, 2015, 2016). Hence proteins were probably first produced by ribosome-free systems, such as non-ribosomal peptide synthesis (Roy and Ibba, 2010; Chen Y. et al., 2016). We propose that when ribosomal protein translation evolved, it used the same punctuation signals as nucleotide polymerization, especially RNA transcription. This assumption yields the testable prediction that signals punctuating DNA/RNA polymerizations are the same as those in protein translation. This second principle assumes that synthesis of nucleotide sequences evolved before translation. Hence, we postulate that punctuation signals for transcription were included into the genetic code’s classical translation system.

We applied this to the example in **Figure 1**. This yields the evolutionary step D, where the sequence signal 5′-GCCGG-3′ used for initiating DNA synthesis after OL-binding could have become the punctuation mark that initiates codon translation. By considering known initiation codons, mainly represented by ATG, the sequence 5′-GCCGG-3′ does not fit any one (Elzanowski and Ostell, 2013). However, this sequence, when translated as codons, codes for alanine and glycine, the structurally simplest amino acids. These were the first integrated in the genetic code according to any hypotheses considering emergence of life (Trifonov, 2000, 2004), and according to protein sequence comparisons (Trifonov, 2009). In this case the sequence 5′-GCCGG-3′, a consensual signal for initiating replication, would reflect, at the translational level, an ancestral coding sequence for initiation of a primitive form of translation.

### Termination Signals for Nucleotide and Amino Acid Synthesis

The nucleotide triplets TAA, TAG, TGA, which function as termination codons in translation, are hotspots for single nucleotide deletions during polymerization (Jestin and Kempf, 1997). Putatively, these frameshift mutation signals could have become translation termination signals.

### Translation Termination: Structural Signal Versus Codon

Translation termination is not always based on stop codons. The genetic code of some ciliates lacks punctuation signals dedicated to the termination of peptide chain elongation (Swart et al., 2016). Similarly, the termination codon may be missing in some mRNAs (Schaub et al., 2012). As mentioned above, tmRNAs sometimes take this termination role. In some cases, translational termination combines secondary and primary structures, as shown in **Figure 1B** for polymerization signals. This principle is in line with an otherwise unexplained observation of the architecture of the genetic code: codon assignments maximize potential for hairpin formation (Itzkovitz and Alon, 2007), and in parallel maximize numbers of off frame stops, which prevent translation after ribosomal frameshifts (Seligmann and Pollock, 2004; Seligmann, 2007, 2010b, 2012a; Tse et al., 2010; Krizek and Krizek, 2012). This link between translation termination and hairpin formation might reflect that current termination codons replaced hairpins.

### Parallels between Transcription and Translation Frameshifts

A further convergence exists between transcription and translation signals, regarding effects of homopolymer nucleotide triplets (AAA, CCC, GGG, TTT). These homopolymers do not confer any information regarding the reading frame (Crick et al., 1957). They induce frameshift mutations, meaning that nucleotides are ‘missed’ during DNA and RNA polymerizations (Atkins et al., 2016). These triplets also cause ribosomal slippage during protein translation (Klobutcher and Farabaugh, 2002; Ketteler, 2012; Advani and Dinman, 2016). In other words, they mark programmed frameshifts for nucleotide polymerizations (DNA replication and RNA transcription) and translation. This classical parallel between nucleotide polymerizations and translation frameshift by homopolymers is tested here on recently described mitochondrial RNAs, so called delRNAs (Seligmann, 2015a, 2016). These RNAs reflect an unusual transcription pattern called del-transcription, in which one or two nucleotides are consistently deleted after each transcribed nucleotide triplet. Here we take advantage of delRNAs to test whether known translational frameshifting signals also function as transcriptional deletion signals. This would enable to test, for frameshifting-deletion signals, the hypothesis that nucleotide polymerization signals were carried over to translation.

### Transcription that Systematically Deletes Nucleotides

The systematic deletions characterizing delRNAs might result from processes including, but not restricted to posttranscriptional editing (Li et al., 2011; Bar-Yaacov et al., 2013; Wang et al., 2014).

Mechanisms regulating del-transcriptions are unknown. We herein tested the hypothesis that detected human delRNAs are enriched in homopolymer nucleotide triplets, as compared to other human mitogenome regions not covered by delRNA. We test a further prediction of the hypothesis that punctuations for DNA/RNA polymerizations were carried over to ribosomal translation of mRNAs into proteins. In this case, we test whether a known system that punctuates ribosomal translation, the natural circular code that enables translation frame retrieval (see below explanations), also regulates systematic mono- and dinucleotide deletions occurring after transcription of nucleotide triplets in mitochondrial delRNA_3-1_ and delRNA_3-2_ (Seligmann, 2015a).

### The Natural Circular Code X

Initiation and termination codons are translational signals that punctuate boundaries of protein coding sequences. The genetic code also includes a little known system of signals that punctuates the coding frame within protein coding sequences, by regulating the ribosomal translation frame. This intra-gene punctuation system was discovered by analyses of protein coding genes that identified one specific set of 20 codons overrepresented in the protein coding frame as compared to the other, non-coding frames of the genes. These 20 codons constitute a circular code (Arquès and Michel, 1996). Here we will test whether this translational circular code also applies to transcription of delRNAs.

Briefly, within the genetic code, circular codes are sets of codons that allow retrieving the frame of any circular word built by these codons (Lacan and Michel, 2001; Fimmel and Strüngmann, 2016; Fimmel et al., 2016). The notion of punctuation of such a word is inherent to codons composing the circular code. This is because any combination of two codons from such a code produces non-redundant text.

Circular codes by definition can not include the four homopolymer nucleotide triplets AAA, CCC, GGG, and TTT that frequently cause frameshifts. Circular codes can only include the 60 remaining codons. For any codon XYZ in a circular code, its permutations YZX and ZXY can not be part of that circular code. Therefore, for any codon included in a circular code, the two codons formed by its permutations can’t be included in that circular code. This means that circular codes include at most 20 codons (60/3). Such circular codes of 20 codons are maximal, and are called ‘maximal circular codes’ because a circular code with more than 20 codons is impossible in the context of the genetic code. Among 60 codons, 3^20^∼349 million potential combinations of 20 codons include ∼ 13 million maximal circular codes.

There are only 221554 maximal C^3^ circular codes. The property C^3^ means that the 20 permutations of the 20 circular code codons XYZ to YZX (example AAC->ACA) form a maximal circular code, and that the 20 permutations of these 20 circular code codons XYZ to ZXY (example AAC->CAA) also form a maximal circular code. There are only 216 circular codes of 20 codons that are self-complementary and have the C^3^ property (Gonzalez et al., 2011; Michel, 2014; Fimmel and Strüngmann, 2015). Self-complementarity means that for any codon of the circular code, its inverse complement occurs among the remaining X codons (example AAC/GTT).

The 20 codons {AAC, AAT, ACC, ATC, ATT, CAG, CTC, CTG, GAA, GAC, GAG, GAT, GCC, GGC, GGT, GTA, GTC, GTT, TAC, TTC} overrepresented within protein coding frames of genes constitute one of these 216 above-defined codes (Arquès and Michel, 1996). Indeed, this set of 20 codons has the following properties: (a) it is a circular code (for any codon XYZ, the 19 remaining codons do not include its permutations YZX and ZXY); (b) it is maximal (20 codons); it has self-complementarity (for any codon, the 19 remaining codons include its inverted complement); and (c) it is C^3^. The latter property means that the permutations YZX and ZXY of XYZ produce each sets of 20 codons that are circular codes. These two circular codes are maximal and C^3^, but lack self-complementarity.

Note that the 20 trinucleotides that Arquès and Michel (1996) detected overrepresented in the +1 and the +2 frames of protein coded genes are indeed permutations ZXY and YZX of the the circular code detected in the coding frame. Obtaining this empirical result within natural gene sequences, considering all possible combinations of 20 codons, has *P* = 6.2 × 10^-8^. These codons code for their assigned amino acid, and also constitute the natural circular code ‘X’, which enables to retrieve the ribosomal translational frame (Arquès and Michel, 1996; Ahmed et al., 2007, 2010; Michel, 2012; El Soufi and Michel, 2014, 2015).

Codons of X code for thirteen of the twenty natural amino acids. X does not include stop codons, nor homopolymers of nucleotide triplets. X enables to detect the ribosomal translation frame, but mechanisms by which this occurs remain unknown. Codons of X can be seen as opposite to homopolymer nucleotide triplets in terms of signaling the translation frame.

The human mitochondrial delRNAs are analyzed here in terms of the circular code X. This is done in order to test whether X regulates del-transcription. We expect that punctuation by X is common to transcription and translation, as predicted in **Figure 1**.

## Materials and Methods

**FIGURE 2:**
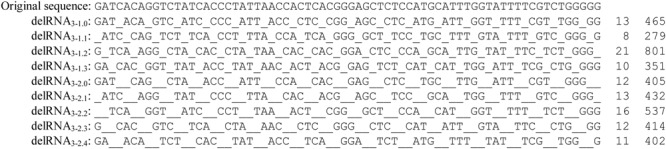
**Systematic deletions after each polymerized trinucleotide, resulting in nine del-transformations of the original sequence.** The first indice number indicates the number of polymerized nucleotides, here three, the second number the deleted nucleotides, followed by the number of nucleotides missing at the 5′ extremity of the original untransformed sequence before the process of systematic deletions starts. The example refers to the first nucleotides of the human mitogenome NC_012920. Each delRNA type is followed by numbers of detected contigs and their total length, as previously described (Seligmann, 2015a, therein Tables 1, 2).

Here we present for the reader’s convenience the methods used by Seligmann (2015a) to detect delRNAs. The human mitochondrial mitogenome NC_012920 was transformed according to systematic mono- and dinucleotide deletions after each trinucleotide. This produces four versions of the mitogenome missing every fourth nucleotide, and five mitogenome versions missing every fourth and fifth nucleotides. **Figure 2** shows these nine transformations for a given sequence.

Note that in principle, systematic deletions might follow patterns that differ from those described in **Figure 2**. Systematic deletions of more than two nucleotides, and after polymerization of less or more than three nucleotides, might exist. This means that delRNAs are characterized by numbers of polymerized nucleotides, followed by numbers of deleted/missing nucleotides. For example, systematic deletions of mononucleotides after each polymerized trinucleotide are noted delRNA_3-1_. Dinucleotide deletions are noted delRNA_3-2_, regular RNAs lacking deletions could be noted in this system delRNA_3-0_. According to this annotation system, the still untested hypothesis of systematic mononucleotide deletions after each polymerized dinucleotide would be noted delRNA_2-1_.

Systematic deletions are characterized by an additional variable, which is the frame of deletion, as compared to the first nucleotide of the regular, untransformed template sequence. Systematic mononucleotide deletions after each polymerized trinucleotide can follow four frames: the process starts at the first nucleotide of the regular, untransformed sequence, polymerizing the three first nucleotides and deleting the fourth, then polymerizing nucleotides five to seven, deleting the eighth, and so on. This case is noted delRNA_3-1.0_. The process can also start at the second, or at the third or the fourth nucleotide, resulting in notations delRNA_3-1.1_, delRNA_3-1.2_, and delRNA_3-1.3_. For systematic dinucleotide deletions after each trinucleotide, corresponding annotations for the five potential deletion frames are delRNA_3-2.0_, delRNA_3-2.1_, delRNA_3-2.2_, delRNA_3-2.3_, and delRNA_3-2.4_ (see the nine del-transformations of a sequence in **Figure 2**).

The nine del-transformations of the human mitogenome were analyzed by blastn (Altschul et al., 1997), by comparing them to 72 samples of human transcripts sequenced by RNA-Seq, Illumina HiSeq 2500 technology, from Genbank’s Sequence Read Archive (SRA), entries SRX768406-SRX768476 (Garzon et al., 2014).

These blast analyzes detected RNAs matching the nine del-transformed versions of the mitogenome, presented by Seligmann (2015a), therein Tables 1, 2 for delRNAs_3-1_ and delRNAs_3-2_, respectively. Here, **Figure 2** indicates the numbers of distinct mitogenome regions (contigs) covered by these RNAs, and the total number of nucleotide sites covered by detected delRNAs, for each of the nine del-transformations examined by Seligmann (2015a). These results from previous analyzes are presented here within this section because they consist the ‘materials’ used here for further analyzes.

We counted numbers of nucleotide triplets for each of the nine del-transformations of the human mitogenome, separately for sequences covered by detected delRNAs, and for the rest of the corresponding del-transformed mitogenome (not covered by detected delRNA). Note that the nucleotide triplets counted are for del-transformed versions of the human mitogenome, not for the original untransformed mitogenome. This count is done only for contiguous nucleotide triplets, meaning nucleotide triplets between deleted nucleotide(s): the triplets do not cover nucleotide(s) deleted from the untransformed mitogenome. Separate counts were done for each of the four homopolymer triplets, and for the 20 nucleotide triplets of the natural circular code X.

We calculate the ratios between frequencies of nucleotide triplets within detected delRNAs and their frequencies in the rest of the human mitogenome (not covered by delRNAs). These ratios indicate whether a specific nucleotide triplet is overrepresented within detected delRNAs, as compared to the rest of the mitogenome when the ratio is >1, or underrepresented when the ratio is <1. We used chi-square statistics to test whether nucleotide triplet counts differ between sequences covered by detected delRNAs, versus the rest of the mitogenome.

## Results

### Excess Homopolymer Nucleotide Triplets in delRNAs

Previous results detected delRNAs for each type of del-transcription (Seligmann, 2015a), meaning RNAs matching the human mitogenome at the condition that one assumes systematic deletions after each transcribed nucleotide triplet. **Figure 2** shows the number of nucleotide sites of the human mitogenome covered by delRNAs, for each delRNA type. Our working hypothesis is that homopolymers contribute to the systematic deletions that produce delRNAs. This would show that del-transcription occurs as a result of signals that are known common to replicational/transcriptional and translational frameshifting, the homopolymer nucleotide triplets. This would put del-transcription in the context of the evolutionary model of carryover of nucleotide polymerization signals to translational signals (**Figure 1**).

**Table 1** compares numbers of homopolymer nucleotide triplets within the deletion frames of detected delRNAs [delRNAs described by Seligmann (2015a), therein Tables 1, 2] and their corresponding number in the same deletion frame in the rest of the mitogenome (for which no delRNA was detected). Detected delRNAs_3-1_ and delRNAs_3-2_ include 1.51 and 2.35 times more homopolymers, respectively, than del-transformed versions of the human mitogenome for which no delRNA was detected. This difference in homopolymer contents between del-transformed mitogenome regions covered by delRNAs, and the rest of the del-transformed mitogenome, is statistically significant for each delRNAs_3-1_ and delRNAs_3-2_ (*P* < 0.001, chi-square tests). Hence, homopolymer nucleotide triplets are significantly associated with delRNAs. This observation that delRNAs are not random sequences, but specifically enriched in triplets that cause frameshifts excludes that delRNA detections result from spurious alignments due to the shear large quantity of transcripts compared with the del-transformed mitogenome. Results confirm the role of homopolymers as transcriptional frameshifting signals in the specific context of systematic deletions during transcription.

**Table 1:** Numbers of homopolymers (AAA, CCC, GGG, TTT) among trinucleotides within del-transformed versions of the human mitogenome, for detected delRNAs (as described by Seligmann, 2015a, therein Tables 1, 2), versus corresponding numbers in remaining human mitogenome regions, assuming the same del-transformation (columns headed by ‘other’).

Trinucleotide	delRNA_3-1_	Other	delRNA_3-2_	Other
AAA	37	487	61	463
CCC	24	600	47	577
GGG	0	72	1	71
TTT	22	229	17	234
All homopol	83	1388	126	1345
Total	632	15934	730	15837
Percent	13.14	8.71	19.94	8.49

*The last line indicates percentages of homopolymer nucleotide triplets in del-transformed sequences. Detected delRNAs have 1.51 and 2.35 times more homopolymers than del-transformed versions of the human mitogenome for which no delRNA has been detected.*

### Nucleotide Triplets in delRNAs that Belong to the Natural Circular Code X

We also analyze delRNA nucleotide triplet contents in relation to a known system signaling the translational frame, the natural circular code X, which regulates ribosomal translation. The evolutionary scenario in **Figure 1** assumes that translational signals (such as the circular code) were carried over from nucleotide polymerizations to translation. This hypothesis predicts non-random associations between delRNAs and the natural circular code X.

We counted codons of X within previously detected delRNAs, and compared their frequency with that in the rest of the human mitogenome, not covered by delRNAs. The total frequency of X codons does not differ between del-transcribed and other sequences.

However, if X has a role in both transcription and translation, these roles may be conflicting for delRNAs. At the level of translation, X should maintain the ribosomal frame, expecting overrepresentation of codons of X in detected delRNAs. For del-transcription, codons of X, if they affect transcription, would prevent transcriptional frameshifts that characterize delRNAs. Hence, X should be underrepresented in delRNAs. Therefore, the lack of bias regarding X in detected human mitochondrial delRNAs could be due to opposite transcriptional versus translational constraints.

### Codon-Specific Reading Frame Retrieval (RFR)

In order to test whether codons of X have opposite roles in the transcription versus translation for delRNAs we apply analyses that differentiate X codons in function of their ability to detect the programmed coding frame. RFR, the reading frame retrieval (RFR) score, estimates for each codon of X its contribution to frame detection, as previously defined (Ahmed et al., 2010; Michel and Seligmann, 2014). For example, triplets ACC and GGT contribute to frame detection in 69% of the cases. CAG, CTC, CTG, and GAG contribute to frame detection in 100% of cases. Their RFR is 69 and 100, respectively; the RFR of the remaining 14 codons is between these two extremes. In the context of delRNAs, we expect that codons with low RFR should be overrepresented and those with high RFR underrepresented if transcription constraints prevail in delRNAs over translation constraints. We expect the opposite if translation constraints prevail over transcriptional ones.

**FIGURE 3:**
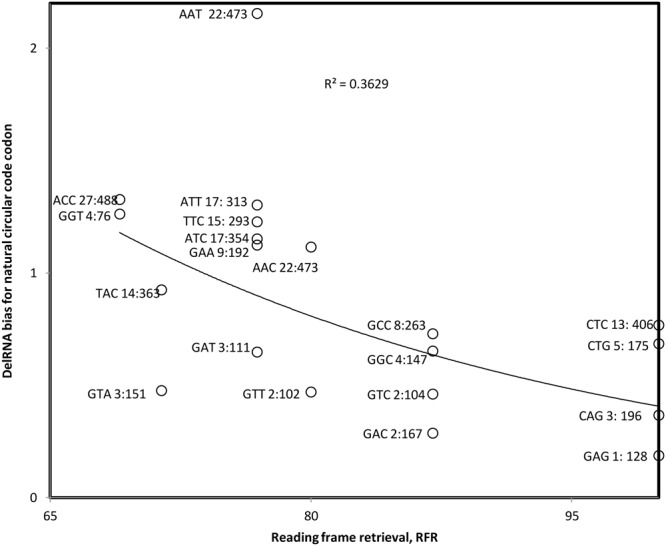
**Bias in natural circular code codon contents in detected delRNA_3-1_ as compared to the rest of the human mitogenome, as a function of the contribution of that codon to RFR.** Natural circular code codon identities are indicated near datapoints, together with numbers of codons within delRNAs, followed by that number in the rest of the del-transformed human mitogenome. The Pearson correlation coefficient *r* = –0.602 (one-tailed *P* = 0.0025) and the nonparametric Spearman rank correlation coefficient *r*s = –0.637 (one tailed *P* = 0.001) indicate that circular code codons with high effects on frame maintenance are avoided.

We calculate the ratio between the frequency of codons of X in delRNAs and their frequency in the rest of the mitogenome. This ratio is plotted as a function of the corresponding RFR for delRNA_3-1_ (**Figure 3**). The Pearson correlation coefficient *r* = –0.602 (one-tailed *P* = 0.0025) and the nonparametric Spearman rank correlation coefficient *r*s = –0.637 (one tailed *P* = 0.001) show avoidance of X codons with high RFR, and overrepresentation of X codons with relatively low RFR. This result is in line with a balance between transcriptional and translational effects of the natural circular code X and suggests that X might affect systematic transcriptional deletions. The pattern in **Figure 3** suggests that codons with high RFR are avoided in delRNAs, to enable transcriptional frameshifting, but that codons of X with low RFR are overrepresented, enabling some relatively weak, yet existing, translational frame regulation. Hence this result indicates a dual role of the natural circular code in del-transcription and the regulation of translation of its products, the delRNAs.

**FIGURE 4:**
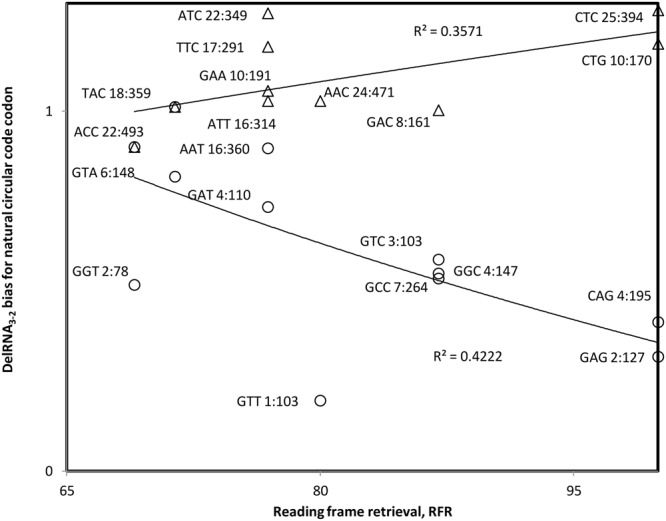
**Bias in natural circular code codon contents in detected delRNA_3-2_ as compared to the rest of the human mitogenome, as a function of the contribution of that codon to reading frame retrieval, RFR.** Natural circular code codon identities are indicated near datapoints, together with numbers of codons within delRNAs, followed by that number in the rest of the del-transformed human mitogenome. Considering all datapoints, the trend indicates avoidance of codons with high RFR (*r* = –0.197, *P* = 0.203; *r*s = –0.097, *P* = 0.342, one tailed tests). Trends seems opposite for codons with bias above versus below ‘1’, suggesting translational frame stabilizing constraints for codons with bias > ‘1’ (*r* = 0.598, *P* = 0.068; *r*s = 0.532, *P* = 0.114, two tailed tests), and transcriptional frameshifting constraints for bias < ‘1’ (*r* = –0.65, *P* = 0.011; *r*s = –0.606, *P* = 0.019, one tailed tests).

We performed the same test for delRNA_3-2_. Results overall confirm those described above that transcription constraints decrease bias for X codons in relation to the RFR of X codons, at least for the underrepresented half of X codons. However, they also indicate that the overrepresented half of X codons follow translational constraints (**Figure 4**). These results that show dual transcriptional and translational roles for the natural circular code are in line with the evolutionary hypothesis that nucleotide polymerization signals were hijacked for translation. They suggest that the natural circular code has its origins in the regulation of nucleotide polymerizations.

## Conclusion

Some examples suggest an evolutionary scenario where DNA punctuation evolved from secondary structures signaling polymerization initiation, termination, and/or processing to linear sequence motifs, which further evolved to translational signals (**Figure 1**). Presumably, primitive low-specificity structural signals evolved into a transition state where both structural and linear nucleotide sequence signals confer high specificity to the punctuation system. Presumably, signals consisting only of linear sequences (evolutionary phase C in **Figure 1**) are more derived and metabolically more efficient because these depend on shorter sequences, but with specificity intermediate between that of the presumed first and the second evolutionary phases. The vertebrate mitochondrial light strand replication origin, OL, and stem-loop hairpins formed by DNA that templates for mitochondrial tRNAs, seem to fit this evolutionary scenario.

Stem-loop hairpins, which punctuate mitochondrial RNA processing (Ojala et al., 1981), also regulate codon-amino acid assignments (Lobanov et al., 2010), suggesting that transcriptional punctuation marks evolved into translational ones. Analyses of mitochondrial delRNA codon content show that codons belonging to the natural circular code X (which regulates ribosomal frame retrieval during translation) affect del-transcription. This first empirical evidence for effects of the natural circular code X on frameshifting deletions during nucleotide synthesis fits the complex predictions of dual functions of X by maintaining translation frame and promoting transcriptional frameshifts. Punctuation signals common to translation and transcription are compatible with the scenario that the former evolved from the latter. Similar scenarios could apply to the evolution of some of the genetic code’s codon-amino acid assignments.

## Author Contributions

HS designed the research and MS, NEH critically revised the MS.

## Conflict of Interest Statement

The authors declare that the research was conducted in the absence of any commercial or financial relationships that could be construed as a potential conflict of interest.
